# Attention-deficit/hyperactivity disorder and risk for psychiatric and neurodevelopmental disorders in siblings

**DOI:** 10.1017/S0033291718000521

**Published:** 2018-04-02

**Authors:** Elina Jokiranta-Olkoniemi, Keely Cheslack-Postava, Petteri Joelsson, Auli Suominen, Alan S. Brown, Andre Sourander

**Affiliations:** 1 Department of Child Psychiatry, University of Turku and Turku University Hospital, Turku, Finland; 2 Department of Psychiatry, Columbia University Medical Center, New York State Psychiatric Institute, New York, New York; 3 Department of Epidemiology, Columbia University Mailman School of Public Health, New York, NY, USA

**Keywords:** ADHD, epidemiology, neurodevelopmental disorders, psychiatric disorders, sibling

## Abstract

**Background:**

Probands with attention-deficit/hyperactivity disorder (ADHD) are at increased risk for several psychiatric and neurodevelopmental disorders. The risk of these disorders among the siblings of probands has not been thoroughly assessed in a population-based cohort.

**Methods:**

Every child born in Finland in 1991–2005 and diagnosed with ADHD in 1995–2011 were identified from national registers. Each case was matched with four controls on sex, place, and date of birth. The full siblings of the cases and controls were born in 1981–2007 and diagnosed in 1981–2013. In total, 7369 cases with 12 565 siblings and 23 181 controls with 42 753 siblings were included in the analyses conducted using generalized estimating equations.

**Results:**

44.2% of the cases and 22.2% of the controls had at least one sibling diagnosed with any psychiatric or neurodevelopmental disorder (risk ratio, RR = 2.1; 95% CI 2.0–2.2). The strongest associations were demonstrated for childhood-onset disorders including ADHD (RR = 5.7; 95% CI 5.1–6.3), conduct and oppositional disorders (RR = 4.0; 95% CI 3.5–4.5), autism spectrum disorders (RR = 3.9; 95% CI 3.3–4.6), other emotional and social interaction disorders (RR = 2.7; 95% CI 2.4–3.1), learning and coordination disorders (RR = 2.6; 95% CI 2.4–2.8), and intellectual disability (RR = 2.4; 95% CI 2.0–2.8). Also, bipolar disorder, unipolar mood disorders, schizophrenia spectrum disorders, other neurotic and personality disorders, substance abuse disorders, and anxiety disorders occurred at increased frequency among the siblings of cases.

**Conclusions:**

The results offer potential utility for early identification of neurodevelopmental and psychiatric disorders in at-risk siblings of ADHD probands and also argue for more studies on common etiologies.

## Introduction

Attention-deficit hyperactivity disorder (ADHD) is characterized by hyperactivity, inattention, and impulsivity (World Health Organization, 1992). ADHD is a highly prevalent neuropsychiatric disorder; the estimated prevalence is approximately 5% (Polanczyk *et al.* 2007) to 7% (Thomas *et al.* 2015). The reported male:female – ratio varies considerably (Nøvik *et al.* 2006; Willcutt, 2012; Joelsson *et al.* 2016); however, the male predominance is a consistent finding across studies. The development of ADHD is thought to be influenced by both genetic and environmental factors in combination with the interaction between these factors (Banerjee *et al.* 2007; Thapar *et al.* 2013).

Most of the studies examining the familial aggregation of psychiatric disorders and ADHD have been conducted among the parents of the offspring (Lindblad *et al.* 2011; Margari *et al.* 2013; McCoy *et al.* 2014; Sanchez-Gistau *et al.* 2015). Those studies which have been extended to cover the full, non-twin siblings of an ADHD proband have generally focused on examining the familial aggregation of ADHD, which is now well established through twin, clinical, and population-based samples (Faraone *et al.* 2005; Chang *et al.* 2013; Chen *et al.* 2016). To date, there exist only two population-based studies examining disorders other than ADHD among the siblings of an ADHD proband (Larsson *et al.* 2013; Skoglund *et al.* 2015). Both studies were conducted in Sweden. A study by Skoglund *et al.* (2015) showed a nearly twofold increased risk of substance abuse disorders among the siblings of an ADHD proband as compared with the controls. Larsson *et al.* (2013) found that the siblings of ADHD probands had an over twofold risk for bipolar disorder and a nearly twofold increased risk for schizophrenia. Despite these studies, there are several questions that need to be addressed. First, because the previous examination included only a limited number of disorders, confined mainly to those diagnosed during adulthood, little is known about the risk for childhood-onset disorders among the full siblings of ADHD probands. This information may have important implications including identifying at-risk individuals among the siblings of children who are diagnosed with ADHD. Second, the role of potential confounding by the diagnosis of parental psychiatric disorders has not been addressed in the previous studies. Third, this may provide evidence informing the concept of a ‘female protective model’. The ‘female protective model’ suggests that females require greater levels of exposure to risk factors for neurodevelopmental disorders such as ADHD than do males in order to manifest these disorders (Rhee & Waldman, 2004; Jacquemont *et al.* 2014; Taylor *et al.* 2016). Here, it would predict greater risk of psychiatric and neurodevelopmental disorders among the siblings of female *v.* male ADHD probands, based on the hypothesis that female probands would have greater exposure to risk factors, at least some of which are shared with their siblings.

Consequently, we sought to: (1) Examine a wide range of different psychiatric and neurodevelopmental disorders among full siblings of probands diagnosed with ADHD; (2) examine these associations stratified by sex of the proband to test whether the siblings of females with ADHD were more likely to themselves exhibit neurodevelopmental disorders than were siblings of males with ADHD.

## Methods

### Data sources

The present study is a part of nationwide ADHD register study, which has been described in detail previously (Joelsson *et al.* 2016). The study is based on a national birth cohort including every live birth between the years 1991 and 2005 (*n* = 900 603) in Finland. Each child was followed up for the diagnosis of ADHD from 1995 to the end of 2011 and matched individually with four controls, as described in more detail below. Data from different nationwide registers were linked using unique personal identity codes, which are given to every newborn in Finland and remain trackable throughout the lifespan. The collection of the data and matching was conducted by the authorities in the Finnish Central Population Register (FCPR). Approval for the use and linkage of health register data was obtained from the Ministry of Social Affairs and Health in Finland. Ethical approval was received from the Ethics Committee of the Hospital District of Southwest Finland.

The three registers used in the study were the following: the Finnish Hospital Discharge Register (FHDR), the Finnish Medical Birth Register (FMBR), and the FCPR.

The FHDR includes computerized national data since 1969 of all medical diagnoses, both somatic and psychiatric, made in hospitals or inpatient wards of local health centers, military wards, and prison hospitals. Starting in 1998, the FHDR includes outpatient care given in public specialized hospital units. Diagnoses are based on the International Classification of Diseases. The Eighth Revision (ICD-8) was used from 1969 to 1986, Ninth Revision (ICD-9) from 1987 to 1995 and Tenth Revision (ICD-10) from 1996 onward. The FHDR was used to identify: (1) children diagnosed with ADHD; (2) cases and controls with severe/profound intellectual disability (ID); (3) diagnoses of psychiatric and/or neurodevelopmental disorders among siblings of the cases, controls, and their parents.

The FMBR, established in 1987, contains comprehensive maternal, fetal, and newborn health-related standardized data on every live birth in Finland collected during pregnancy until 7 days of age. Starting in 1990, it also includes data on maternal occupational status classified into the following categories: upper white-collar workers (e.g. teachers, leaders, and experts); lower white-collar workers (lower clerical workers e.g. office workers, who are not leaders or experts); blue-collar workers (perform manual labor); and others (entrepreneurs and people outside the labor force, e.g. students, homemakers, and unemployed). The FMBR was used to obtain the data on covariates, as discussed below.

The FCPR is a computerized national register, maintained by the Finnish population center and local register offices. It contains basic population data such as name, personal identity code, address, native language, family relationships, and deaths of every Finnish citizen and people residing permanently in Finland. The FCPR was used to identify: (1) controls, (2) parents, (3) full siblings, (4) and siblings who have died or emigrated before age three.

### Identification of cases, controls, and their siblings

**Fig. 1.**
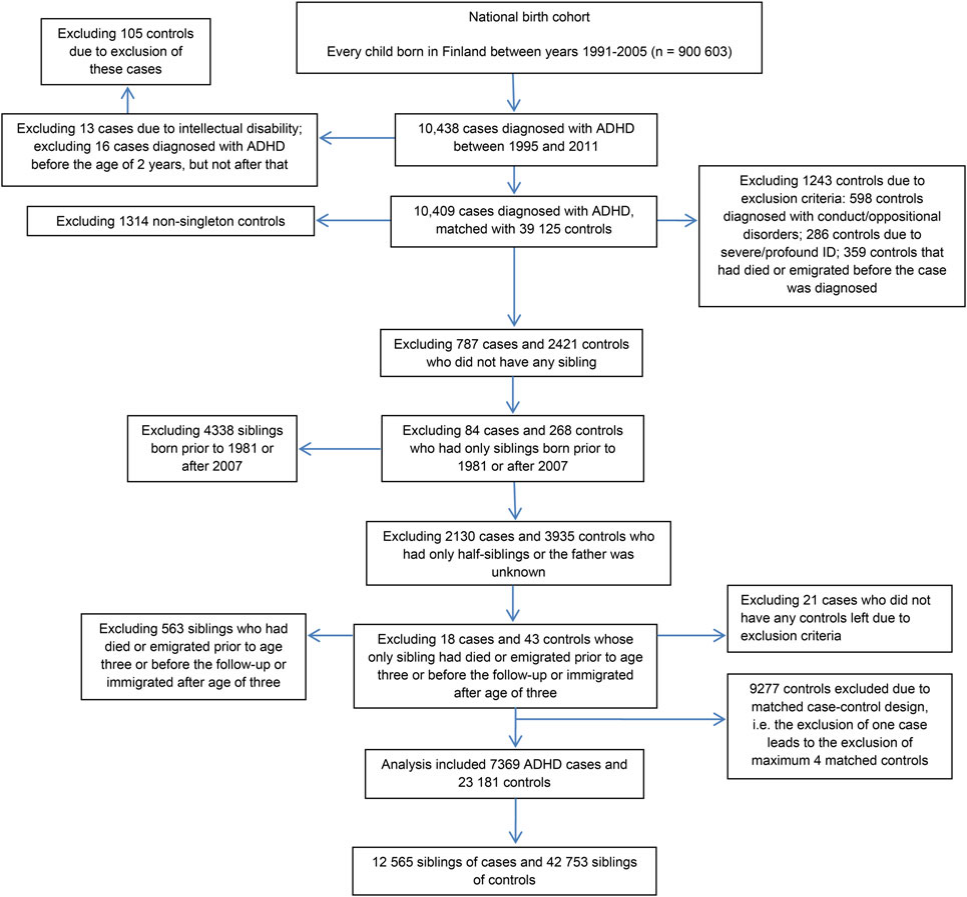
The selection of the study population.

The selection of the study sample including cases, controls, and their siblings is presented in Fig. 1. Every child born in Finland between 1991 and 2005, and diagnosed with ADHD between 1995 and 2011, was identified from the FHDR using ICD-9 (314x) and ICD-10 (F90.x) codes. The most recent diagnosis was used and therefore every case was diagnosed using ICD-10 codes. We excluded 16 children who were registered with a diagnosis of ADHD only prior to the age of 2 years. Moreover, 13 children with additional diagnoses of profound or severe ID were excluded, because the reliability of ADHD diagnosis might be uncertain among the children with severe or profound ID.

Each case was individually matched with four controls on sex, date of birth (±30 days) and place of birth, as identified from the FCPR. Matching factors were selected due to sex-bias in the disorder and to control for potential confounding by unmeasured spatial and temporal factors. Controls were excluded if they: died or emigrated from Finland before the case was diagnosed; or if they had a diagnosis of ADHD, conduct/oppositional disorder, or profound/severe ID, based on data from the FHDR. The exclusion of controls with conduct/oppositional disorders was made because these subjects may have been misdiagnosed ADHD patients. This exclusion is likely to have had only a minor influence on the results given that the cumulative incidence of conduct/oppositional disorders by the age of 14 in Finland is only 1.4% (Gyllenberg *et al.* 2014).

The full siblings of cases and controls were identified from the FCPR, and their diagnoses of psychiatric and/or neurodevelopmental disorders were identified from the FHDR. Siblings were born between 1981 and 2007, and diagnosed by the end of 2013. This was done so that both older and younger siblings were included and the youngest siblings had at least 6 years follow-up for the diagnosis. Cases and controls without any full sibling were excluded. As shown in the Fig. 1, a total of 7369 cases with 12 565 full siblings and 23 181 controls with 42 753 full siblings were included in the analysis.

### Psychiatric and neurodevelopmental diagnosis among the siblings

The specific diagnoses presented in the online Supplementary Appendix were each classified using a dichotomous variable (no/yes). Pooled diagnostic classes including *any psychiatric/neurodevelopmental disorder* and *any disorder usually diagnosed in childhood* (referred to as *any childhood-onset disorder*) were classified using dichotomous variables (no/yes). In the case of multiple disorders in a sibling (e.g. anxiety and eating disorder), the sibling was considered as positive for all of them.

### Covariates

Covariates included in the analysis were measured factors hypothesized to be potential confounders: matching variables, i.e. sex, date of birth, and place of birth (Sjölander & Greenland, 2013); maternal age (Chudal *et al.* 2014, 2015); paternal age (Chudal *et al.* 2014, 2015); maternal psychiatric disorder (Sucksdorff *et al.* 2014; Joelsson *et al.* 2017); paternal psychiatric disorder (Sucksdorff *et al.* 2014; Joelsson *et al.* 2017); maternal immigration status (Lehti *et al.* 2015, 2016); paternal immigration status (Lehti *et al.* 2015, 2016); maternal socioeconomic status (SES) (Costello *et al.* 2003; Larsson *et al.* 2014); and maternal parity (Haukka *et al.* 2004; Marín *et al.* 2014). We also adjusted for the birth years of the siblings to make sure that the results were not influenced by different birth year distributions among the siblings of cases *v.* controls. Data on birth years of the siblings and parents and immigration status were gathered from the FCPR, parental psychiatric diagnoses from the FHDR, and maternal SES and parity from the FMBR. Covariate data are presented in online Supplementary Table S1.

### Statistical analysis

Bivariate analysis using the Pearson's χ^2^ test was conducted to assess the significance of associations between the covariates and (1) any psychiatric or neurodevelopmental disorders in the siblings, among controls only, and (2) ADHD. A Pearson's χ^2^ test was also conducted to examine possible differences in sibship sizes between the cases and matched controls.

In our primary analyses, the dependent variable was the status of the ADHD case or control, and the independent variable was a dichotomous (no/yes) measure of the psychiatric or neurodevelopmental disorder in the sibling. We estimated the association between ADHD status in probands and psychiatric or neurodevelopmental disorders in the siblings, separately for each disorder investigated, by using generalized estimating equations with an exchangeable correlation matrix. The method was applied to account for the dependence between cases and matched controls and the siblings within the same family. We adapted regression analyses with a log-link function (Liang & Zeger, 1986). Follow-up years were used as an offset to account for the length of follow-up of siblings in the estimation of model parameters. Each stratum included the siblings of a single case and of each matched control.

The first model (model I) was adjusted for matching variables; the second model (model II) was adjusted for matching variables and all covariates except for maternal and paternal psychiatric disorder; the final model (model III) was adjusted for matching variables and all covariates. The modeling was first based on all ADHD probands and then stratified by sex of the proband. The interaction term was assessed to evaluate the significance of the risk ratios (RRs) for disorders in siblings between females with ADHD *v.* males with ADHD and their sex-matched controls. An additional analysis excluded every sibling diagnosed with ADHD to verify that the results were not due solely to ADHD among siblings.

The relation between ADHD status in the proband and the sibling outcome was reported as RRs with 95% confidence intervals (CIs) together with the *p* values. Associations with two-sided *p* values with *p* < 0.05 were considered statistically significant. The statistical analyses were conducted by using SAS statistical software (SAS Institute Inc. SAS Version 9.4. Cary, NC; 2010).

## Results

All covariates tested, except maternal immigrant status, were associated with any psychiatric or neurodevelopmental disorder among the siblings and with ADHD case status (Supplementary Table S1). The number of the siblings among ADHD cases and matched controls are presented in Table 1. As shown in the table, cases were more likely to have a smaller sibship as compared with the controls (*p* < 0.0001); 56% of the cases *v.* 52% of controls had only one sibling (Table 1).

**Table 1.** Number of siblings among ADHD cases and matched controls in the nationwide birth cohort^a^

Number of siblings	Case	Control
1	4123 (56.0%)	12 048 (52.0%)
2	2181 (29.6%)	7392 (31.9%)
3 or more	1065 (14.5%)	3741 (16.1%)
Mean (SD)	1.7 (1.1)	1.8 (1.4)
Range	1–17	1–17
Median (IQR)	1 (1–2)	1 (1–2)

ADHD, attention-deficit hyperactivity disorder; IQR, interquartile range; SD, standard deviation.

a Cases born in 1991–2005, diagnosed with ADHD in 1995–2011, full siblings born in 1981–2007, diagnosed in 1981–2013.

Table 2 shows the frequencies and adjusted RRs in all three models for different diagnostic categories among the siblings of the cases and controls. Statistically significant results remained in all three models, even though the strongest associations were observed in the first model. For the sake of brevity, only the results from the final model (model III) are reported herein. As shown in Table 2 in pooled diagnostic classes, 44.2% of the cases had at least one sibling diagnosed with any psychiatric or neurodevelopmental disorder. Among the controls, the corresponding figure was 22.2% (RR = 2.1; 95% CI 2.0–2.2, *p* < 0.001). The increased risk was also demonstrated for childhood-onset disorders: 36.7% of the cases had at least one sibling diagnosed with any childhood-onset disorder, *v.* 15.3% of the controls (RR = 2.5; 95% CI 2.4–2.6, *p* < 0.001) (Table 2). Increased risk among the siblings of the cases was demonstrated for every disorder examined except eating disorders. The strongest risks were observed for ADHD (RR = 5.7; 95% CI 5.1–6.3, *p* < 0.001), conduct and oppositional disorders (RR = 4.0; 95% CI 3.5–4.5, *p* < 0.001), and ASD (RR = 3.9; 95% CI 3.3–4.6, *p* < 0.001). Over twofold increased risks were demonstrated for other emotional and social interaction disorders (RR = 2.7; 95% CI 2.4–3.1, *p* < 0.001), learning and coordination disorders (RR = 2.6; 95% CI 2.4–2.8, *p* < 0.001), tic disorders (RR = 2.5; 95% CI 1.9–3.3, *p* < 0.001), and ID (RR = 2.4; 95% CI 2.0–2.8, *p* < 0.001). Siblings of the cases were also at an increased risk for bipolar disorder (RR = 1.9; 95% CI 1.4–2.6, *p* < 0.001), unipolar mood disorders (RR = 1.9; 95% CI 1.7–2.0, *p* < 0.001), schizophrenia spectrum disorders (RR = 1.8; 95% CI, 1.5–2.3, *p* < 0.001), other neurotic and personality disorders (RR = 1.7; 95% CI 1.4–2.0, *p* < 0.001), substance abuse disorders (RR = 1.7; 95% CI 1.5–2.0, *p* < 0.001), and anxiety disorders (RR = 1.4; 95% CI 1.3–1.6, *p* < 0.001) as compared with the siblings of the controls.

**Table 2.** Associations between ADHD and psychiatric and neurodevelopmental disorders among the siblings of cases and matched controls

	ADHD^a^		Adjusted^b^ (model I)		Adjusted^c^ (model II)		Adjusted^d^ (model III)	
	Case *n* = 7369 (%)	Control *n* = 23 181 (%)	RR	(95% CI)	RR	(95% CI)	RR	(95% CI)
ADHD	1134 (15.4)	598 (2.6)	7.1	(6.4–7.8)***	6.5	(5.9–7.2)***	5.7	(5.1–6.3)***
Conduct and oppositional disorders	705 (9.6)	461 (2.0)	5.6	(5.0–6.3)***	5.0	(4.5–5.7)***	4.0	(3.5–4.5)***
ASD	322 (4.4)	264 (1.1)	4.6	(3.9–5.4)***	4.4	(3.7–5.2)***	3.9	(3.3–4.6)***
Other emotional and social interaction disorders	533 (7.2)	530 (2.3)	3.7	(3.3–4.2)***	3.4	(3.0–3.9)***	2.7	(2.4–3.1)***
Learning and coordination disorders	1538 (20.9)	1928 (8.3)	3.0	(2.8–3.2)***	2.8	(2.6–3.0)***	2.6	(2.4–2.8)***
Tic disorders	93 (1.3)	123 (0.5)	2.7	(2.0–3.5)***	2.6	(2.0–3.5)***	2.5	(1.9–3.3)***
Intellectual disability	226 (3.1)	278 (1.2)	2.8	(2.4–3.4)***	2.5	(2.1–3.0)***	2.4	(2.0–2.8)***
Bipolar disorder	82 (1.1)	124 (0.5)	2.2	(1.7–3.0)***	2.3	(1.7–3.0)***	1.9	(1.4–2.6)***
Unipolar mood disorders	906 (12.3)	1445 (6.2)	2.2	(2.1–2.4)***	2.2	(2.0–2.4)***	1.9	(1.7–2.0)***
Schizophrenia spectrum disorders	147 (2.0)	249 (1.1)	2.1	(1.7–2.5)***	2.1	(1.7–2.6)***	1.8	(1.5–2.3)***
Other neurotic and personality disorders	218 (3.0)	391 (1.7)	2.0	(1.7–2.3)***	2.0	(1.7–2.4)***	1.7	(1.4–2.0)***
Alcohol and drug addiction/abuse	316 (4.3)	513 (2.2)	2.1	(1.8–2.4)***	2.0	(1.8–2.3)***	1.7	(1.5–2.0)***
Anxiety disorders	503 (6.8)	1050 (4.5)	1.7	(1.5–1.9)***	1.6	(1.5–1.8)***	1.4	(1.3–1.6)***
Eating disorders	111 (1.5)	344 (1.5)	1.2	(0.9–1.4)	1.2	(0.97–1.5)	1.1	(0.9–1.4)
Pooled diagnostic classes								
Any psychiatric/neurodevelopmental disorder	3254 (44.2)	5154 (22.2)	2.4	(2.3–2.5)***	2.3	(2.2–2.4)***	2.1	(2.0–2.2)***
Any childhood-onset disorder	2703 (36.7)	3557 (15.3)	2.9	(2.8–3.1)***	2.7	(2.6–2.9)***	2.5	(2.4–2.6)***

ADHD, attention-deficit hyperactivity disorder; ASD, autism spectrum disorders; CI, confidence interval; RR, risk ratio.

* = *p* < 0.05, ** = *p* < 0.01, *** = *p* < 0.001.

a Cases born in 1991–2005, diagnosed with ADHD in 1995–2011, full siblings born in 1981–2007, diagnosed in 1981–2013.

b Adjusted for sex, birth date, and birth place.

c Adjusted for sex, birth date, birth place, birth year of siblings, maternal and paternal age, paternal immigrant status, maternal parity, and socioeconomic status.

d Adjusted for sex, birth date, birth place, birth year of siblings, maternal and paternal age, maternal and paternal psychiatric disorder, paternal immigrant status, maternal parity, and socioeconomic status.

Table 3 shows the adjusted associations among the siblings of the cases and controls, stratified by the sex of the proband. Among the siblings of male probands, ADHD was associated with every disorder examined except eating disorders. Among the siblings of female probands, the associations were observed in every disorder category except for bipolar disorder and eating disorders. Based on interaction analysis, the siblings of the female case probands were at greater risk for any psychiatric/neurodevelopmental disorder (*p* = 0.03), any childhood-onset disorder (*p* = 0.02), and from the specific diagnostic categories with ID (*p* = 0.01) and learning and coordination disorders (*p* *<* 0.01) as compared with the siblings of male case probands.

**Table 3.** Associations between ADHD and psychiatric and neurodevelopmental disorders among the siblings of male and female cases and controls

	ADHD^a^ male				ADHD^a^ female			
	Male case *n* = 6213 (%)	Male control *n* = 19 520 (%)	Adjusted^b^ (model II) RR (95% CI)	Adjusted^c^ (model III) RR (95% CI)	Female case *n* = 1156 (%)	Female control *n* = 3661 (%)	Adjusted^b^ (model II) RR (95% CI)	Adjusted^c^ (model III) RR (95% CI)
ADHD	916 (14.7)	505 (2.6)	6.2 (5.5–6.9)***	5.5 (4.9–6.1)***	218 (18.9)	93 (2.5)	8.2 (6.3–10.6)***	6.6 (5.0–8.7)***
Conduct and oppositional disorders	576 (9.3)	385 (2.0)	5.0 (4.4–5.7)***	4.0 (3.5–4.6)***	129 (11.2)	76 (2.1)	5.3 (4.0–7.1)***	3.7 (2.7–5.0)***
ASD	262 (4.2)	228 (1.2)	4.2 (3.5–5.0)***	3.7 (3.1–4.5)***	60 (5.2)	36 (1.0)	6.0 (4.0–9.2)***	4.7 (3.1–7.3)***
Other emotional and social interaction disorders	440 (7.1)	445 (2.3)	3.4 (3.0–3.9)***	2.7 (2.4–3.1)***	93 (8.0)	85 (2.3)	3.7 (2.7–5.1)***	2.5 (1.8–3.6)***
Learning and coordination disorders	1231 (19.8)	1612 (8.3)	2.7 (2.5–2.9)***	2.5 (2.3–2.7)***	307 (26.6)	316 (8.6)	3.5 (3.0–4.1)***	3.1 (2.7–3.7)***
Tic disorders	78 (1.3)	104 (0.5)	2.6 (1.9–3.6)***	2.5 (1.8–3.4)***	15 (1.3)	19 (0.5)	NA	NA
Intellectual disability	168 (2.7)	235 (1.2)	2.3 (1.8–2.7)***	2.1 (1.7–2.6)***	58 (5.0)	43 (1.2)	4.0 (2.7–6.0)***	3.5 (2.3–5.3)***
Bipolar disorder	70 (1.1)	96 (0.5)	2.5 (1.8–3.4)***	2.1 (1.5–2.9)***	12 (1.0)	28 (0.8)	1.5 (0.8–3.1)	1.2 (0.6–2.4)
Unipolar mood disorders	757 (12.2)	1221 (6.3)	2.2 (2.0–2.4)***	1.9 (1.7–2.1)***	149 (12.9)	224 (6.1)	2.2 (1.8–2.7)***	1.7 (1.4–2.2)***
Schizophrenia spectrum disorders	117 (1.9)	208 (1.1)	2.1 (1.6–2.6)***	1.8 (1.4–2.3)***	30 (2.6)	41 (1.1)	2.4 (1.5–4.0)***	1.9 (1.1–3.1)*
Other neurotic and personality disorders	177 (2.9)	326 (1.7)	1.9 (1.6–2.3)***	1.6 (1.4–2.0)***	41 (3.6)	65 (1.8)	2.2 (1.5–3.2)***	1.8 (1.2–2.6)**
Alcohol and drug addiction/abuse	267 (4.3)	440 (2.3)	2.0 (1.7–2.3)***	1.7 (1.5–2.0)***	49 (4.2)	73 (2.0)	2.1 (1.5–3.0)***	1.8 (1.2–2.6)**
Anxiety disorders	415 (6.7)	887 (4.5)	1.6 (1.4–1.8)***	1.4 (1.2–1.6)***	88 (7.6)	163 (4.5)	1.8 (1.4–2.3)***	1.4 (1.1–1.9)**
Eating disorders	88 (1.4)	296 (1.5)	1.1 (0.9–1.4)	1.0 (0.8–1.3)	23 (2.0)	48 (1.3)	1.9 (1.2–3.0)*	1.7 (0.99–2.8)
Pooled diagnostic classes								
Any psychiatric/neurodevelopmental disorder	2676 (43.1)	4350 (22.3)	2.2 (2.2–2.3)***	2.0 (2.0–2.1)***	578 (50.0)	804 (22.0)	2.6 (2.4–2.9)***	2.3 (2.1–2.5)***
Any childhood-onset disorder	2217 (35.7)	3003 (15.4)	2.7 (2.5–2.8)***	2.4 (2.3–2.6)***	486 (42.0)	554 (15.1)	3.2 (2.9–3.6)***	2.8 (2.5–3.1)***

ADHD, attention-deficit hyperactivity disorder; ASD, autism spectrum disorders; CI, confidence interval; RR, risk ratio; NA, not applicable.

* = *p* < 0.05, ** = *p* < 0.01, *** = *p* < 0.001.

*Note*: Bolded RRs refer to the results of the interaction analysis showing the statistically significant difference between the siblings of male probands *v.* siblings of female probands.

a Cases born in 1991–2005, diagnosed with ADHD in 1995–2011, full siblings born in 1981–2007, diagnosed in 1981–2013.

b Adjusted for sex, birth date, birth place, birth year of siblings, maternal and paternal age, paternal immigrant status, maternal parity, and socioeconomic status.

c Adjusted for sex, birth date, birth place, birth year of siblings, maternal and paternal age, maternal and paternal psychiatric disorder, paternal immigrant status, maternal parity, and socioeconomic status.

The adjusted associations after excluding the siblings diagnosed with ADHD are presented in the online Supplementary Table S2. A similar pattern was seen as in the main analysis, i.e. ADHD was associated with every disorder investigated except for eating disorders.

## Discussion

This is the first population-based study to examine a wide range of psychiatric and neurodevelopmental disorders among the siblings of ADHD probands. The first main finding is an increased risk for every disorder examined except eating disorders among the siblings of the cases as compared with the siblings of the controls. The strongest risks were observed with ADHD, conduct and oppositional disorders and ASD. The second main finding is that the associations were stronger among the siblings of female *v.* male probands.

The strongest associations were observed with the disorders that are typically diagnosed in childhood. The findings are novel because previous population-based studies have focused on only a few, specific disorders. The non-specific pattern, i.e. almost all disorders are associated with ADHD, seen in this study is consistent with increasing evidence showing familial aggregation of psychiatric disorders, with onsets varying from childhood to adulthood (Song *et al.* 2015; Jokiranta-Olkoniemi *et al.* 2016). This observed pattern is also congruent with genome-wide association studies reporting an overlap of genetic factors across several psychiatric disorders including ADHD (Cross-Disorder Group of the Psychiatric Genomics Consortium, 2013*a*, *b*). Nevertheless, the hypothesis of a shared genetic origin of different disorders (O'Donovan & Owen, 2016; Pettersson *et al.* 2016) does not exclude the possibility of common environmental risk factors shared by family members. Given that we examined only biological siblings, environmental factors shared by the siblings could be related, for example, to prenatal factors that are common to the mother during pregnancies of different offspring. In any case, these results further challenge our understanding of traditional diagnostic boundaries based on current and past classification systems. To be able to clarify these hypotheses of shared genetic and environmental factors, further genetic and family studies including those of twins and half-siblings are needed.

Our results show similarities in the magnitude and direction of associations with the previous population-based studies which have examined the familial clustering of ADHD (Chen *et al.* 2016), substance abuse disorders (Skoglund *et al.* 2015) and psychotic disorders (Larsson *et al.* 2013). The study extends our understanding about the familial clustering of these disorders by showing that the associations persist after adjusting for the diagnosis of these disorders among the parents. Moreover, as ADHD frequently co-occurs with substance abuse disorders (van Emmerik-van Oortmerssen *et al.* 2012) and psychotic disorders (Larsson *et al.* 2013), by excluding the siblings with ADHD, we were able to show that the associations observed were not due to comorbidity of these disorders among the siblings. Previous studies have shown that the risk for these disorders among relatives increases with increasing genetic relatedness to an ADHD proband (Larsson *et al.* 2013; Skoglund *et al.* 2015; Chen *et al.* 2016), further supporting a role for genetic factors in the development of these disorders.

Siblings of ADHD females were in general at greater risk for different psychiatric and childhood-onset disorders than the siblings of males: Half of the female cases (50%) had at least one sibling diagnosed with any disorder while among ADHD males the corresponding figure was 43%. For childhood-onset disorders, 42% of the female cases had at least one sibling diagnosed *v.* 36% of the male cases. Among specific disorder categories examined, the increased risk in siblings of female probands was observed for ID and learning and coordination disorders. The higher prevalence of ADHD as well as other neurodevelopmental disorders seen among males has been hypothesized to indicate that the female sex would somehow protect females from neurodevelopmental disorders (Jacquemont *et al.* 2014). This hypothesis has gained some tentative support in a few twin studies (Rhee & Waldman, 2004; Taylor *et al.* 2016), but not in population-based studies other than the present one (Chen *et al.* 2016; Jokiranta-Olkoniemi *et al.* 2016; Ghirardi *et al.* 2018). An alternative explanation for the results observed in the present study could be that females with moderate or less severe ADHD symptoms are underrepresented: inattentive symptoms are more typical among females (Nussbaum, 2012) and more easily go unrecognized than do hyperactive symptoms, which are more common among males (Gershon, 2002; Yoshimasu *et al.* 2012). Consequently, diagnosed ADHD females may have a more severe symptom profile, which may reflect the greater burden of different genetic and environmental risk factors at the family level (Auerbach *et al.* 2017). Thus, this load could also be seen among siblings with a greater burden of disorders.

The ages of the siblings ranged from 6 to 32 at the end of follow-up, and only a subset had passed through the age of risk for adult-onset disorders. Therefore, it is probable that additional cases of these disorders will eventually be diagnosed among the siblings of cases and controls. These low numbers may have reduced the precision of our estimates. However, this would be expected to have biased the estimates of association from our study only if the identification of outcomes occurred differentially between cases and controls. For example, if siblings of ADHD cases experience onset or diagnosis of disorders at an earlier age than do siblings of controls, this would have biased our estimates for these disorders upwards. While we cannot rule it out, this suggests that it is unlikely that the associations for adult disorders were underestimated to a large extent.

The strengths of the study are the use of a national birth cohort including comprehensive register data on 900 603 children together with their biological parents and siblings and the inclusion of several covariates. Consequently, our data are unlikely to be biased by reporting differences (e.g. recall bias), selective ascertainment, or loss to follow-up. There are also limitations. First, as the data is based on Finnish registers, subjects’ psychiatric disorders were not directly verified. However, several diagnoses including ADHD, ASD, tic disorders, schizophrenia, and bipolar disorder have been shown to have good validity in the FHDR (Isohanni *et al.* 1997; Kieseppä *et al.* 2000; Perälä *et al.* 2007; Lampi *et al.* 2010; Sund, 2012; Leivonen *et al.* 2014; Joelsson *et al.* 2016). Second, the FHDR includes subjects diagnosed in specialized services, not in primary healthcare, and therefore likely represents subjects with at least moderately severe conditions. Thus, as the coverage of moderate and severe neurodevelopmental/psychiatric disorders is high in the FHDR, less severe disorders treated only in primary healthcare are likely to be underestimated. We expect, however, that our data provide good coverage of subjects with moderate and severe psychiatric and neurodevelopmental conditions because the Finnish healthcare services are universal and financed by the state and municipalities, which do not hinder utilization of treatment services. Finally, the siblings of cases may differ in treatment seeking as compared with the siblings of controls. This is unlikely, however, because in Finland virtually every child is comprehensively assessed at least once a year in the municipal child health clinics before they start school at age seven. Concern about possible neurodevelopmental or other disorders raised in these regular health check-ups leads to referral for specialized healthcare services.

## Conclusions

We have demonstrated, in a large, national sample that the risks of different childhood and adult-onset psychiatric and neurodevelopmental disorders are elevated among the siblings of probands diagnosed with ADHD. These findings suggest that ADHD shares familial and environmental vulnerability factors with these other disorders and are consistent with studies suggesting familial aggregation of ADHD and other psychiatric disorders. The greater family-level burden of psychiatric and neurodevelopmental disorders among ADHD females require further research. These results may be used to improve early identification of at-risk siblings based on a proband diagnosis of ADHD and also argue for more studies on common etiologies.

## Supplementary material

For supplementary material accompanying this paper visit https://doi.org/10.1017/S0033291718000521. click here to view supplementary material
