# Supplementary material for: Attention-deficit/hyperactivity disorder and risk for psychiatric and neurodevelopmental disorders in siblings
Source: Psychol Med. 2018 Apr 2;49(1):84–91. doi: 10.1017/S0033291718000521 (PMC6316367; doi:10.1017/S0033291718000521)
Supplement: Supplementary file 1 [file S0033291718000521sup001.zip › S0033291718000521sup001/Supplement Appendix.doc]

| **Supplement Appendix. Diagnostic classification of mental disorders according to the ICD-10, ICD-9 and ICD-8 diagnoses.** | | | |
| --- | --- | --- | --- |
|  | **ICD-10** | **ICD-9** | **ICD-8** |
| Attention-deficit hyperactivity disorder | F90 | 314 |  |
| Conduct and oppositional disorders | F91, F92 | 3120A, 3123C, 3123D, 3138A | 308.99 |
| Autism spectrum disorders | F84 | 299 |  |
| Other emotional and social interaction disorders | F93, F94 | 3092A, 3092B, 3133A |  |
| Learning and coordination disorders | F80-83 | 315 | 306.00, 306.10, 306.30 |
| Tic disorders | F95 | 3072A, 3072B, 3072C, 3072D | 306.20 |
| Intellectual disability | F70–F79 | 317-319 | 310-315 |
| Bipolar disorder | F30, F31 | 2962A-G, 2963A-G, 2964A-G, 2967A |  |

| Unipolar mood disorders | F32, F33, F34, F38, F39 | 2961A-G, 2968A, 3004A, 2988A |  |
| --- | --- | --- | --- |
| Schizophrenia and other nonaffective psychoses (referred as schizophrenia spectrum disorders) | F20, F21, F22, F23, F24, F25, F28, F29 | 295, 297, 2989X, 3012C |  |
| Other neurotic and personality disorders and other nonpsychotic disorders (referred as other neurotic and personality disorders) | F41.2, F43, F44 F45, F48, F51 (excluding F51.3; F51.4), F52, F53, F54, F55, F59, F60, F62, F63, F65, F66, F68, F69, F99 | 300–302 (excluding 3000A, 3000B, 3000C, 3002B, 3002C, 3002D, 3002X, 3003A, 3004A and 3012C), 3074A, 3074F, 3074H, 3075C, 3075E, 3078A, 3079X, 309 (excluding 3092A and 3092B), 312 (excluding 3120A, 3123C and 3123D) |  |
| Alcohol and drug addiction/abuse | F10-19 | 303–305, 291–292 |  |
| Anxiety disorders | F40, F41, (excluding F41.2), F42 | 3000A, 3000B, 3000C, 3002B, 3002C, 3002D, 3002X, 3003A |  |
| Eating disorders | F50 | 3071A, 3075A, 3075B |  |
| **Pooled diagnostic classes** |  |  |  |

| Any psychiatric/ neurodevelopmental disorder | F10-F99 | 291-319, excluding 316 |  |
| --- | --- | --- | --- |
| Any disorder usually diagnosed in childhood (referred as any childhood onset disorder) | F70-F79, F80, F81, F82, F83, F84, F88, F89, F90, F91, F92, F93, F94, F95, F98, F51.3, F51.4 | 299, 313, 314, 315, 317-319, 3120A, 3123C, 3123D, 3070A, 3070B, 3072A, 3072B, 3072C, 3072D, 3073A, 3074G, 3075D, 3076A, 3076B, 3076C, 3077A, 3092A, 3092B | 306.00, 306.10, 306.20, 306.30, 306.60, 306.70, 308.99, 310–315 |
