# Supplementary material for: Attention-deficit/hyperactivity disorder and risk for psychiatric and neurodevelopmental disorders in siblings
Source: Psychol Med. 2018 Apr 2;49(1):84–91. doi: 10.1017/S0033291718000521 (PMC6316367; doi:10.1017/S0033291718000521)
Supplement: Supplementary file 1 [file S0033291718000521sup001.zip › S0033291718000521sup001/Supplementary Table S1 final.doc]

| **Supplementary Table S1. Covariates in relation to psychiatric/neurodevelopmental disorders among the siblings of controls and in relation to ADHD.** | | | | |
| --- | --- | --- | --- | --- |
|  | Presence of psychiatric/neurodevelopmental disorders among the siblings, controls only | | | Association between covariates and ADHD |
|  | No disorder, n **(%)** | Yes, n **(%)** | P | P |
| **Maternal age (years)** |  |  | < .0001 | < .0001 |
| ≤24 | 3,063 **(17.0)** | 968 **(18.8)** |  |  |
| 25-34 | 12,534 **(69.5)** | 3,327 **(64.6)** |  |  |
| ≥35 | 2,430 **(13.5)** | 859 **(16.7)** |  |  |
| **Paternal age (years)** |  |  | < .0001 | < .0001 |
| ≤24 | 1,544 **(8.6)** | 477 **(9.3)** |  |  |
| 25-34 | 11,765 **(65.3)** | 3,091 **(60.0)** |  |  |
| ≥35 | 4,718 **(26.2)** | 1,586 **(30.8)** |  |  |
| **Maternal psychiatric disorder*** |  |  | < .0001 | < .0001 |
| No | 16,373 **(90.8)** | 4,256 **(82.6)** |  |  |
| Yes | 1,654 **(9.2)** | 898 **(17.4)** |  |  |
| **Paternal psychiatric disorder*** |  |  | < .0001 | < .0001 |
| No | 16,277 **(90.3)** | 4,276 **(83.0)** |  |  |
| Yes | 1,750 **(9.7)** | 878 **(17.0)** |  |  |
| **Maternal immigration status** |  |  | 0.78 | < .0001 |
| No | 17,805 **(98.8)** | 5,088 **(98.7)** |  |  |
| Yes | 222 **(1.2)** | 66 **(1.3)** |  |  |
| **Paternal immigration status** |  |  | 0.04 | <.0001 |
| No | 17,750 **(98.5)** | 5,054 **(98.1)** |  |  |
| Yes | 277 **(1.5)** | 100 **(1.9)** |  |  |
| **Maternal socioeconomic status**** |  |  | < .0001 | < .0001 |
| Upper white collar workers | 2,975 **(16.5)** | 640 **(12.4)** |  |  |
| Lower white collar workers | 8,350 **(46.3)** | 2,147 **(41.7)** |  |  |
| Blue collar workers | 2,885 **(16.0)** | 986 **(19.1)** |  |  |
| Others | 2,554 **(14.2)** | 1,009 **(19.6)** |  |  |
| Missing | 1,263 **(7.0)** | 372 **(7.2)** |  |  |
| **Maternal parity** |  |  | < .0001 | < .0001 |
| First born child | 6,648 **(37.1)** | 1,254 **(24.5)** |  |  |
| Second or later born | 11,265 **(62.9)** | 3,866 **(75.5)** |  |  |
| **Birth year** |  |  | < .0001 | < .0001 |
| 1981 | 235 (0.64) | 40 (0.66) |  |  |
| 1982 | 328 (0.89) | 41 (0.67) |  |  |
| 1983 | 433 (1.18) | 58 (0.95) |  |  |
| 1984 | 505 (1.38) | 85 (1.39) |  |  |
| 1985 | 576 (1.57) | 96 (1.58) |  |  |
| 1986 | 703 (1.92) | 116 (1.90) |  |  |
| 1987 | 867 (2.37) | 173 (2.84) |  |  |
| 1988 | 1135 (3.10) | 213 (3.50) |  |  |
| 1989 | 1610 (4.39) | 280 (4.59) |  |  |
| 1990 | 1689 (4.61) | 370 (6.07) |  |  |
| 1991 | 1766 (4.82) | 362 (5.94) |  |  |
| 1992 | 1846 (5.04) | 427 (7.01) |  |  |
| 1993 | 1961 (5.35) | 480 (7.88) |  |  |
| 1994 | 2281 (6.22) | 464 (7.61) |  |  |
| 1995 | 2278 (6.21) | 456 (7.48) |  |  |
| 1996 | 2284 (6.23) | 423 (6.94) |  |  |
| 1997 | 2169 (5.92) | 383 (6.28) |  |  |
| 1998 | 1985 (5.41) | 326 (5.35) |  |  |
| 1999 | 2009 (5.48) | 258 (4.23) |  |  |
| 2000 | 1820 (4.96) | 250 (4.10) |  |  |
| 2001 | 1653 (4.51) | 224 (3.68) |  |  |
| 2002 | 1512 (4.12) | 160 (2.63) |  |  |
| 2003 | 1397 (3.81) | 155 (2.54) |  |  |
| 2004 | 1237 (3.37) | 86 (1.41) |  |  |
| 2005 | 961 (2.62) | 77 (1.26) |  |  |
| 2006 | 772 (2.11) | 57 (0.94) |  |  |
| 2007 | 647(1.76) | 34 (0.56) |  |  |
| Abbreviations: ADHD, Attention-deficit hyperactivity disorder  * ICD-10 codes F10-F99, excluding F70-F79; ICD-9 291-316 excluding 293-294; ICD-8 291-309 excluding 292-294  ** Based on maternal occupation at birth. In case of reporting education instead of occupation, a mother is classified as upper white-collar workers if she has reported to have a university degree and as lower white-collar workers if she is known to have a vocational degree lower than university level. | | | | |
