# Supplementary material for: Attention-deficit/hyperactivity disorder and risk for psychiatric and neurodevelopmental disorders in siblings
Source: Psychol Med. 2018 Apr 2;49(1):84–91. doi: 10.1017/S0033291718000521 (PMC6316367; doi:10.1017/S0033291718000521)
Supplement: Supplementary file 1 [file S0033291718000521sup001.zip › S0033291718000521sup001/Supplementary Table S2 final.doc]

| **Supplementary Table S2. Associations between ADHD and psychiatric and neurodevelopmental disorders among the siblings of cases and controls, after exclusion of siblings diagnosed with ADHD^a^** | | | | | | |
| --- | --- | --- | --- | --- | --- | --- |
|  | **Frequencies (%)** | | **Adjusted^b^** | | **Adjusted^c^** | |
|  | Case  n = 6,854 (%) | Control  n = 21,351 (%) | RR | (95 % CI) | RR | (95 % CI) |
| Conduct and oppositional disorders | 409 **(6.0)** | 334 **(1.6)** | 4.1 | (3.5-4.7) *** | 3.2 | (2.8-3.8) *** |
| ASD | 190 **(2.8)** | 193 **(0.9)** | 3.5 | (2.9-4.3) *** | 3.2 | (2.6-3.9) *** |
| Other emotional and social interaction disorders | 346 **(5.1)** | 446 **(2.1)** | 2.7 | (2.4-3.2) *** | 2.2 | (1.9-2.6) *** |
| Learning and coordination disorders | 1,038 **(15.1)** | 1,547 **(7.3)** | 2.3 | (2.1-2.5) *** | 2.2 | **(**2.0-2.4) *** |
| Tic disorders | 51 (0.7) | 89 (0.4) | 2.0 | (1.4-2.8) *** | 1.9 | (1.4-2.7) *** |
| Intellectual disability | 168 **(2.5)** | 235 **(1.1)** | 2.3 | (1.9-2.8) *** | 2.1 | (1.7-2.6) *** |
| Bipolar disorder | 58 **(0.9)** | 104 **(0.5)** | 1.9 | (1.4-2.6) *** | 1.7 | (1.2-2.3) ** |
| Unipolar mood disorders | 744 **(10.9)** | 1,274 **(6.0)** | 2.0 | (1.9-2.2) *** | 1.8 | (1.6-1.9) *** |
| Schizophrenia spectrum disorders | 123 **(1.8)** | 218 **(1.0)** | 2.1 | (1.7-2.6) *** | 1.8 | (1.4-2.3) *** |
| Other neurotic and personality disorders | 184 **(2.7)** | 351 **(1.6)** | 1.9 | (1.5-2.2) *** | 1.6 | (1.3-1.9) *** |
| Alcohol and drug addiction/ abuse | 268 **(3.9)** | 456 **(2.1)** | 1.9 | (1.6-2.2) *** | 1.7 | (1.5-2.0) *** |
| Anxiety disorders | 424 **(6.2)** | 940 **(4.4)** | 1.6 | (1.4-1.8) *** | 1.4 | (1.2-1.6) *** |
| Eating disorders | 94 **(1.4)** | 306 **(1.4)** | 1.2 | (0.9-1.5) | 1.1 | (0.8-1.4) |
| **Pooled diagnostic classes** |  |  |  |  |  |  |
| Any psychiatric/ neurodevelopmental disorder | 2,429 **(35.4)** | 4,361 **(20.4)** | 2.0 | (1.9-2.1) *** | 1.8 | (1.7-1.9) *** |
| Any childhood onset disorder | 1,813 **(26.5)** | 2,834 **(13.3)** | 2.3 | (2.1-2.4) *** | 2.1 | (2.0-2.2) *** |
| Abbreviations: ADHD, Attention-deficit hyperactivity disorder; ASD, Autism spectrum disorders; CI, confidence interval; RR, risk ratio *= p< 0.05, **= p< 0.01, ***= p< 0.001 ^a^Cases born in 1991-2005, diagnosed with ADHD in 1995–2011, full siblings born in 1981-2007, diagnosed in 1981-2013 ^b^Adjusted for sex, birth date, birth place, birth year of siblings, maternal and paternal age, paternal immigrant status, maternal parity and socioeconomic status ^c^Adjusted for sex, birth date, birth place, birth year of siblings, maternal and paternal age, maternal and paternal psychiatric disorder, paternal immigrant status, maternal parity and socioeconomic status | | | | | | |
